# Biomaterials for Periodontal and Peri-Implant Regeneration

**DOI:** 10.3390/ma14123319

**Published:** 2021-06-15

**Authors:** Leonardo Mancini, Mario Romandini, Adriano Fratini, Lorenzo Maria Americo, Saurav Panda, Enrico Marchetti

**Affiliations:** 1 Department of Life, Health and Environmental Sciences, University of L’Aquila, 67100 L’Aquila, Italy; Adriano.fratini@graduate.univaq.it (A.F.); Lorenzomaria.americo@gmail.com (L.M.A.); Enrico.marchetti@univaq.it (E.M.); 2 ETEP Research Group (Etiology and Therapy of Periodontal and Peri-implant Diseases), University Complutense, 28040 Madrid, Spain; mario.romandini@gmail.com; 3 Department of Periodontics and Oral Implantology, Institute of Dental Sciences, Siksha O Anusandhan (Deemed to Be) University, Bhubaneswar 759146, Odisha, India; drsaurav87@gmail.com; 4 Department of Biomedical, Surgical and Dental Sciences, University of Milan, 20122 Milan, Italy

**Keywords:** tissue engineering, periodontal regeneration, biomaterials, 3D printing, growth factors, regeneration

## Abstract

Periodontal and peri-implant regeneration is the technique that aims to restore the damaged tissue around teeth and implants. They are surrounded by a different apparatus, and according to it, the regenerative procedure can differ for both sites. During the last century, several biomaterials and biological mediators were proposed to achieve a complete restoration of the damaged tissues with less invasiveness and a tailored approach. Based on relevant systematic reviews and articles searched on PubMed, Scopus, and Cochrane databases, data regarding different biomaterials were extracted and summarized. Bone grafts of different origin, membranes for guided tissue regeneration, growth factors, and stem cells are currently the foundation of the routinary clinical practice. Moreover, a tailored approach, according to the patient and specific to the involved tooth or implant, is mandatory to achieve a better result and a reduction in patient morbidity and discomfort. The aim of this review is to summarize clinical findings and future developments regarding grafts, membranes, molecules, and emerging therapies. In conclusion, tissue engineering is constantly evolving; moreover, a tailor-made approach for each patient is essential to obtain a reliable result and the combination of several biomaterials is the elective choice in several conditions.

## 1. Introduction

The improved quality and expectancy of life of the current population leads to an increase in injuries and bone disease in older people who have a diminished capacity to restore and regenerate the damaged tissues [1]. Oral and craniofacial tissue injuries are still a very challenging situation for dentists and oral surgeons. To facilitate dentists in their clinical practice, tissue engineering is in constant evolution, and each year several biomaterials are proposed to achieve better results in periodontal and peri-implant regeneration [2]. The principal target of periodontal and peri-implant tissue engineering is to regenerate the supporting tissue of the teeth or implants. Tooth loss, bone, and soft tissue remodeling are consequences of an inflammatory process or age-related decay [3,4]. Periodontitis has been estimated in about 27% of the global population, and to restore missing tooth implants, therapy is a primary alternative to mobile prosthesis [5,6,7]. This condition leads the clinician to evaluate the predisposition of bone and supporting tissue around the missing tooth site. In several conditions, this required a regenerative approach before or during the implant insertion [8,9]. Peri-implantitis has been estimated in 20% of the population and is defined as an inflammatory process that occurs around implants with soft tissue inflammation and supporting bone loss [10].

**Figure 1:**
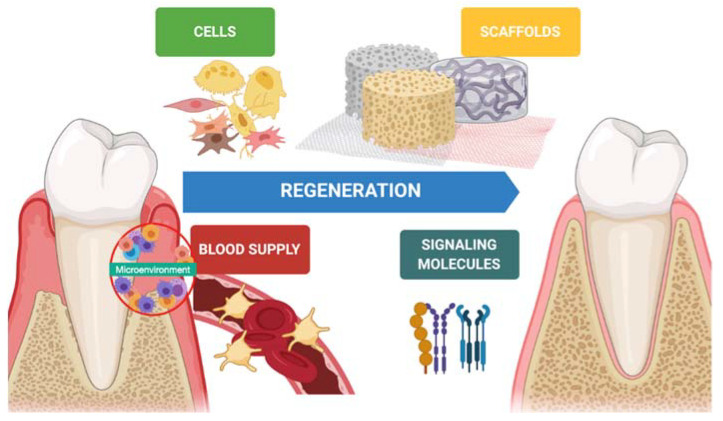
Iconographic representation of the factors involved in periodontal regeneration.

In the last decades, hard tissue regeneration has reached good outcomes regarding newly formed bone, mineralization, and osteoinduction [11]. On the other hand, soft tissue regeneration has also gained interest in preventing advanced forms of periodontitis, peri-implantitis, and mucogingival disorders [12]. Indeed, keratinized tissue, tissue thickness, and supracrestal tissue height around teeth and implants is desirable to achieve better esthetic outcomes and guarantee long-term stability [13,14]. Periodontal regeneration is one of several disciplines that has benefitted from tissue engineering. Biomaterials (scaffolds), molecules (growth factors), and stem cells are keys in the regenerative process, and a synergy between them improves the quality and predictability of the technique (Figure 1).

**Figure 2:**
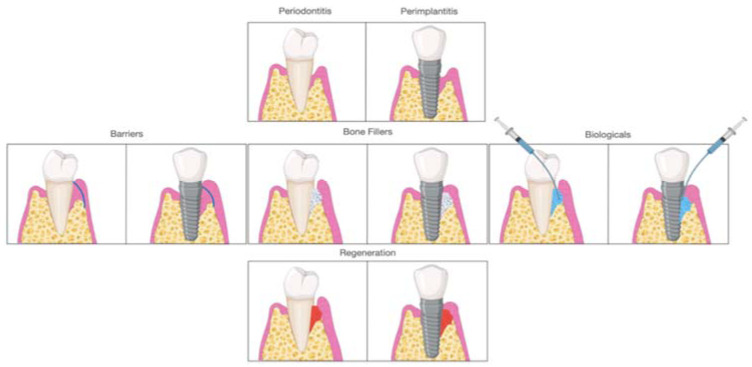
Approaches for regenerating periodontal and peri-implant sites after treating the disease.

Early on, periodontal regeneration used the concept of guided tissue regeneration (GTR), selecting cell populations to colonize the periodontal wound following surgical exposure [15]. The use of bone substitutes in conjunction with barriers aims to prevent epithelial migration. This allows the periodontal ligament cells (PDL) to populate the protected site, providing positive effects in particular cases [16,17]. Decades of research have expanded from this concept, and different biomaterials are available to clinicians and researchers for alveolar bone regeneration. According to the mechanism of action, biomaterials are classified as barriers, bone fillers, and biologicals. In several situations, such as GTR, treatment components are not used alone but always in combination. Barriers are materials that cover the periodontal defect, protecting them from epithelial downgrowth. Bone fillers are scaffolds or bone grafts that replace the missing portion of the alveolar bone [18]. Biologics are growth factors, cell therapy, or substances that can be directly administrated in the defect (Figure 2).

Efforts have been made over recent years to stimulate bone and soft tissue regeneration around teeth as well as edentulous areas and around implants affected by peri-implantitis [19,20,21]. This review aimed to highlight new frontiers regarding periodontal and peri-implant regeneration with a perspective on the application of biomaterials and emerging therapies.

## 2. Materials

Grafting materials are commonly used in periodontal tissue engineering to restore the alveolar bone proper, providing adequate regeneration and tooth stability over the years, or the soft and hard tissues around teeth and implants for clinical and esthetic reasons [22]. They include biological and synthetic materials in various shapes and forms, such as granules, particles, gel, 3D scaffolds, injectable substances, polymers, and matrices. According to the type of regeneration and technique, these materials are used alone or in combinations to improve and accentuate the regenerative process. The followed biomaterials were classified and selected according to the scientific evidence extracted from systematic reviews and articles searched on PubMed, Scopus, and Cochrane databases with the following keywords: “Periodontal regeneration” AND/OR “Biomaterials” OR “Peri-implant regeneration” AND “Bone grafts” AND/OR “Biologics” OR “Stem cells”.

### 2.1. Bone Fillers

**Figure 3:**
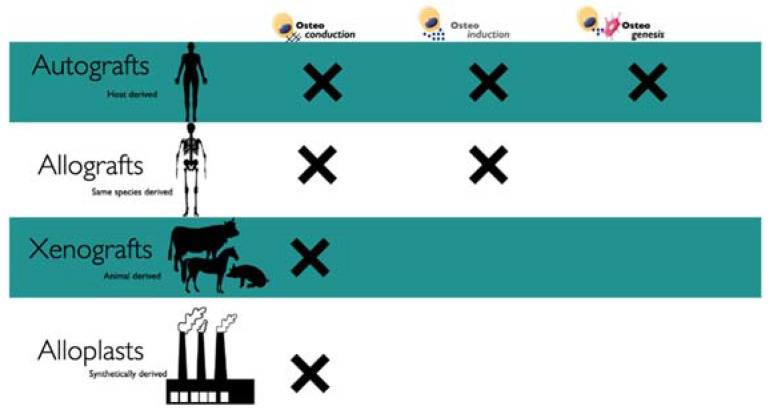
Characteristic of bone grafts according to the origin.

Bone fillers are used in ridge preservation or augmentation and to restore the missing volume of periodontal defects; the presence of teeth or implants next to the defect is important for the regenerative potential. These types of graft are classified according to their origin (Figure 3).

**Figure 4:**
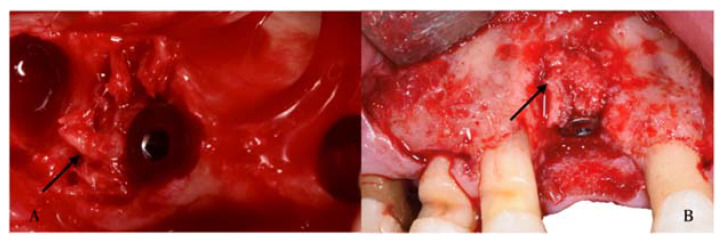
Examples of autografts in the peri-implant site. (**A**) Autologous bone grafts in residual defect after post extractive implant, (**B**) autologous bone grafts on implant after peri-implantitis.

- Autografts are the gold standard due to the osteogenic, osteoconductive, and osteoinductive potential and the absence of foreign body reactions (FBR) [23]. Depending on the size of the defect, the autograft is usually harvested intraorally from the extraction socket, edentulous ridge, symphysis, tuberosity, or buccal plate (Figure 4).

In the presence of large defects, the harvesting process is obtained from extraoral sites, such as the tibia, skull, or iliac crest [24]. These types of interventions require a second site surgery, which increases stress and discomfort for the patient. In using autografts, there are advantages, such as osteogenic potential and reduced risk of disease transmission, but at the same time, the second site surgery includes risk of possible complications, postoperative pain, and limited availability make this approach less attractive for large sites. In the history of periodontal regeneration, the use of autografts was suggested firstly by Nabers and O’Leary in 1965; they proposed the use of cortical bone chips removed manually from within the surgical site [25]. Cushing, in 1969, suggested the use of grafts from the iliac crest for the induction of new bone in the periodontium [26,27]. According to this study, a complete regeneration and furcation coverage was achieved, but nowadays, due to the minimally invasive approach, the presence of several biomaterials is not widespread [28]. In the implant field, autogenous bone is a gold standard. Indeed, the implant survival rate for implants surrendered by autografts, reported in the literature, is between 70–100% [29,30]. The worst records were for the iliac crest with high resorption and implant failure [31]. Data are controversial for some studies that reported similar results between intra and extraoral grafts [32,33].

**Figure 5:**
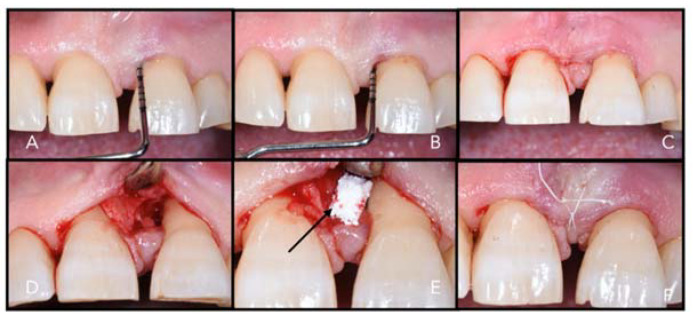
Example of xenografts in a periodontal defect. (**A**) Probing depth, (**B**) bone sounding, (**C**) flap design, (**D**) periodontal defect, (**E**) collagenated bovine bone graft, (**F**) primary wound closure.

- Allografts are biological materials harvested from the same species. The advantage of allografts is the elimination of a second surgical site and tissue availability. Tissue banks are involved in the extraction process from which tissue is extracted, and depending on the treatment, it is possible to obtain freeze-dried bone (FDBAs) or decalcified freeze-dried bone (DFDBAs). The disadvantage in using these types of biomaterials is the possible FBR and disease transmission; although, in the last years, severe and rigid procedures have been developed to reduce the risk [34,35]. Nevertheless, researchers and clinicians identified allografts as reliable sources for the regenerative procedure since they can serve as osteoconductive or osteoinductive biomaterials preserved of proteins in their matrix [36]. The allograft’s decalcification leads to an exposure of bone morphogenic proteins (BMPs) that are effective molecules in bone regeneration [36]. In the case of allograft, the exposure of BMPs showed an increase in bone resorption during the follow-up period. Nevertheless, a disadvantage in using allografts is the high cost compared to xenografts and autografts [37]. Moreover, it is not available in several counties for ethical and legal reasons.
- Xenografts are bone substitutes obtained from other species, such as bovine or porcine grafts, and transplanted into humans. The main disadvantage of xenografts is the antigenicity; indeed, these tissues need to be carefully treated to remove the organic components [18]. Several commercial products have been proposed based on this protocol (Table 1), such as Geistlich Bio-Oss^®^ particles (Geistlich Pharma, Wolhusen, Switzerland), which are harvested bovine and is considered a global reference product in oral regeneration (Figure 5). Despite positive results from several studies, the disadvantage is in the unpredictable grade of regeneration and resorption. The advantages are a single surgical procedure, availability, and reduced patient morbidity. According to Stavropoulos et al. (2005, 2010), the use of deproteinized bovine bone (DBB) in adjunct to GTR renders the defect more stable on a long-term follow-up [38,39,40].

### 2.2. Barriers

After the exposure and debridement of a periodontal defect, several types of cells can recolonize the lesion, such as epithelial cells, which have a fast turnover, fibroblasts cells, or bone and PDL cells [51]. Barriers are used to limit and select cells allowing the regeneration of a specific tissue, such as bone or PDL, reducing the downgrowth of epithelial cells in the defect and not allowing the formation of a long junctional epithelium [52]. Moreover, barriers are used to maintain the space of the defect, facilitating cell replication and tissue regeneration [52]. Traditionally, barriers are divided into resorbable and non-resorbable (needing a second phase for removal). Otherwise, it is possible to classify these matrixes according to their origin, including autogenous, xenogeneic, allogenic, and alloplastic. The efficacy of several membranes was discussed in two articles from Kao et al. (2015) and Sculean et al. (2015) in which positive and negative effects were analyzed [53,54]. The most used in periodontal regeneration are xenogeneic membranes (bovine or porcine origin) enriched with collagen and characterized by a cross or no cross-linked process that can reduce or improve the resorption time. According to Garcia et al. (2017), no differences were achieved regarding guided bone regeneration (GBR) in using cross or no cross-linked matrices; however, regarding tissue integration and postoperative complications, the cross-linked membranes seem to be less predictable [55]. The first type of membrane used in 1980 was based on cellulose acetate, and since then, different types of materials have been developed and studied [52].

#### 2.2.1. Resorbable Barriers

The demand for resorbable barriers during the last decade increased due to a reduction in the numbers of surgeries and biomaterial resorption. The main positive factors that need to be considered are a reduction in patient discomfort, bioactive properties, and the ease of handling [56]. Unfavorable factors are the unpredictable resorption pattern related to the degradation process (hydrolytic or enzymatic) and the possible presence of inflammation related to the degradation process [57]. The raw material may be natural or synthetic, and natural is more biocompatible but with an unpredictable resorption pattern. On the other hand, synthetics have a predictable degradability and a mechanical resistance that is customized according to the production process [58]. The most used and widespread is the collagen harvested, as said before, from animals. Type I collagen is responsible for the attraction and activation of PDL cells and fibroblasts. Thus, it is one of the most used for membrane production (Table 2) [59].

**Figure 6:**
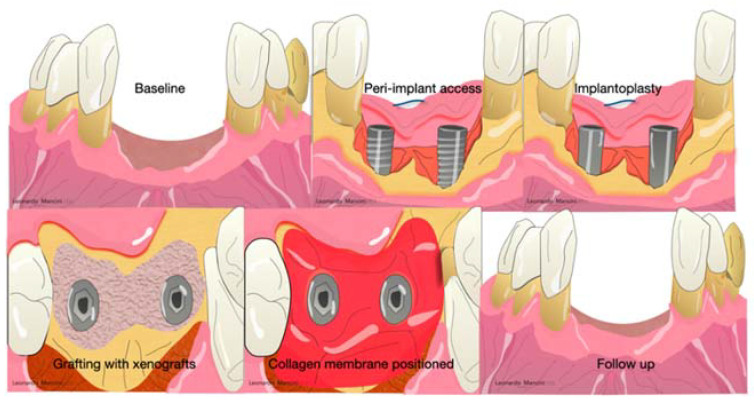
Example of peri-implant regeneration after peri-implantitis with xenografts and collagen membranes. After the peri-implant access, a heavy implant debridement was achieved and an implantoplasty was mandatory to remove the infected surface. The peri-implant defect was filled with xenogeneic grafts and covered with a resorbable membrane.

Collagen membranes are used not only in periodontal regeneration but also in peri-implant regeneration and, in several cases, regenerative procedures associated with an implantoplasty or heavy decontamination of the implant surfaces (Figure 6).

#### 2.2.2. Non-Resorbable Barriers

The main advantages of non-resorbable membranes are the high mechanical stability and the cell’s migration inhibition [56]. However, there are some criticisms, such as the second surgical intervention possible exposure, and accentuated inflammation in case of infection [56]. The most widespread and used during the last decade were polytetrafluoroethylene (ePTFE) and titanium-reinforced membranes [60]. PTFE was developed by Gore-Tex (W. L. Gore & Associates, Inc., Newark, Denmark) in 1990. The particularities were the presence of a double layer with different functions; the first layer is porous, and the aim is to promote cell ingrowth. The other side acts as a space provider to inhibit epithelial cell downgrowth. Several randomized clinical trials showed interesting results after three months of healing in periodontal regeneration [61,62,63,64,65,66]. Others reported several complications (exposure, suppuration, pain) probably related to the flap handling and suture collapsing [65]. Nowadays, these barriers are not used due to the introduction of minimally invasive approaches (minimally invasive surgical technique, single flap approach, or modified minimally invasive approach) that can achieve periodontal regeneration without the selection of cells but with the use of growth factors inside the defect associated with a minimal flaps design that maintains the space in favor of blood clot stability [66,67,68,69,70]. Moreover, with these techniques, the handling of a membrane is not easy to obtain.

### 2.3. Biologics

Biological mediators are considered the last innovation in oral regeneration. It is possible to classify these mediators in stem cells, growth factors, and gene therapy. The most used and widespread are platelet-rich growth factors (PDGF), bone morphogenetic proteins (BMP), and enamel matrix derivatives (EMD).

**Figure 7:**
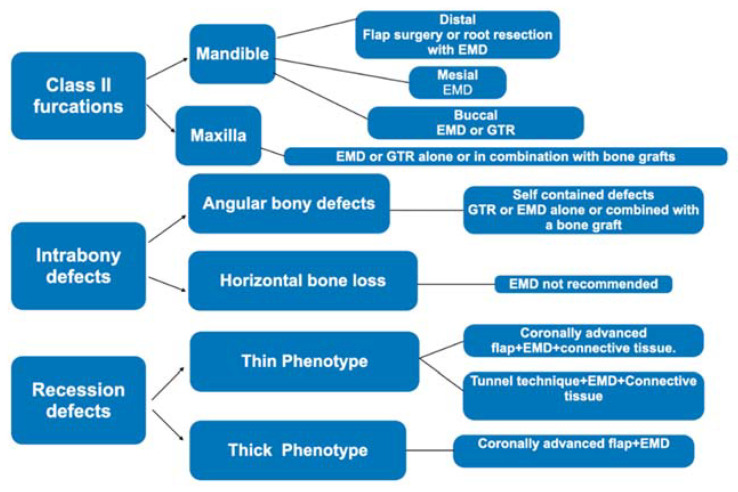
Clinical indications for EMD in periodontal regeneration with respect to the defect’s type modified from Miron et al. [95].

**Figure 8:**
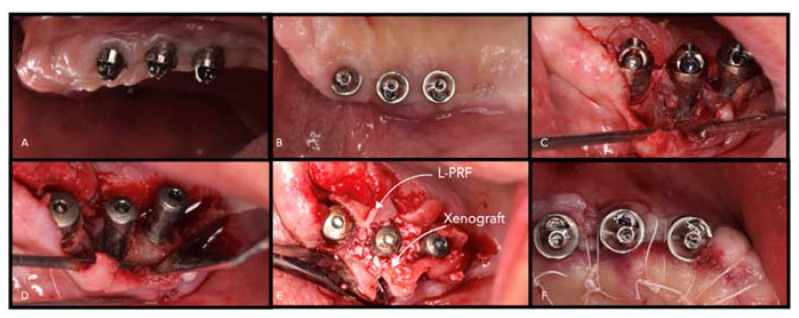
Peri-implant defects treated with L-PRF and xenografts. (**A**) Baseline, (**B**) baseline occlusal view, (**C**) access flap, (**D**) implantoplasty, (**E**) L-PRF membranes and xenografts in the defect, (**F**) primary wound healing.

- PDGF is primarily involved in wound healing; several studies showed its function and ability to enhance the proliferation and migration of PDL cells [71,72]. Moreover, the chemotactic effect leads to a promotion of collagen synthesis and can stimulate gingival fibroblasts to the hyaluronate synthesis [73,74,75,76]. This growth factor might be effective alone or in combination with other growth factors, such as the insulin-like growth factor-1 (IGF-1). Indeed, several in vivo studies showed the efficacy of PDGF in periodontal regeneration alone or combined, and it always demonstrated the new formation of cementum and the production of collagen [77,78,79]. Thanks to molecular cloning, it is now possible to reproduce a recombinant human PDGF [77]. Nevertheless, this type of recombinant product is not sold in several nations, such as Italy, for ethical problems. The most used and analyzed product is GEM 21S^®^, (Osteohealth, Shirley, NY, USA) with in vivo and in vitro studies [80].
- BMPs are factors that belong to the superfamily of transforming growth factor-beta (TGF-ß), are abundant in bone tissue, and are produced by several cells including osteoclasts and osteoblasts. Two types (BMP-4 and BMP-7) are commonly enclosed in allografts, demonstrating osteoinductivity and influencing cells’ behavior in bone regeneration [81,82,83]. Moreover, BMPs act as a chemoattractant for osteoblast precursors and undifferentiated stem cells (MSCs) through the activation of genes related to bone formation, such as osteocalcin [84,85]. A disadvantage in the extraction of BMPs is the synthetic production, which is very expensive, and there is a limitation for the encapsulation in synthetic biomaterials [85].
- EMD is released by Hertwig’s cells during the formation of teeth and periodontal tissue, and these proteins are situated on the root surface, influencing the initial steps of cementum, alveolar bone, and periodontal ligament formation [86,87]. In origin (1996), a Swedish factory (Biora, Malmö, Sweden) released the actual and unique EMD derivatives extracted from porcine enamel in the form of purified acid. Later, Straumann AS acquired the title and Emdogain^®^ (Straumann AG, Basel, Switzerland) is the name of the unique enamel derivates on the market. It is composed mainly of amelogenins, which are specific proteins fundamental in the enamel mineralization process. In physiological conditions, the amelogenins are nano formed, and during the enzymatic degradation by metalloproteinases (MMP), they release bioactive peptides for weeks [88]. In this process, there are advantages, such as the stimulation of new bone and wound healing conditioning. On the other hand, this process might create root resorption due to the presence of MMP and an inflammatory pattern during the regenerative phase [89]. The advantage of using EMD is the mimic action, which can recruit cementoblasts to form new root cementum and consequently facilitate the formation of a new periodontal ligament [89]. This product has been on the market since 1997, and several articles underlined the ease of handling, an interesting result in periodontal regeneration [90,91,92,93,94]. Miron et al. in (2016) collected all the data regarding EMD in periodontal regeneration, and in this study, the use of EMD was relevant in adjunct to non-surgical therapy and regenerative procedures, according to the defect size and shape (Figure 7) [95]. According to the literature, EMD, after 25 years from its introduction, seems to be unique in demonstrating a histological periodontal regeneration with new cementum and periodontal ligament and the presence of Sharpey’s fibers in the periodontal structure [95]. Regarding the use of EMD around implants, data collected from a randomized clinical trial, according to Isehed et al. (2016), revealed that EMD delivered promising but insufficient regeneration associated with an alteration of the Gram-negative flora [96].
- Hyaluronic acid (HA) is a natural glycosaminoglycan contained in several tissues, such as connective tissue. It is an excellent scaffold for periodontal regeneration. Moreover, it seems to have an antimicrobial and anti-inflammatory effect [97,98]. The principal factor that makes this a promising biomaterial is the viscoelastic property and the capacity for absorbing a considerable amount of water. This renders hyaluronic acid a periodontal filler, and, in several situations, it has a protective function as a barrier for bacteria and viruses. Pilloni et al. (2019) suggested the use of HA with a collagen membrane in periodontal defects [99,100]. A systematic review from Eliezer et al. suggested that the addition of HA to non-surgical and surgical periodontal therapy may have additional clinical effects on the clinical attachment level (CAL, 0.73 mm; 95% CI, 0.28 to 1.17 mm; *p* < 0.0001), periodontal depth (PD, 0.36 mm; 95% CI, −0.54 to −0.19 mm; *p* < 0.0001), and bleeding on probing (BoP, 5%; 95% CI, −22 to −8%; *p* < 0.001) [101]. Regarding the use of HA in peri-implant defects, several studies suggest the benefit in microflora diversity, and at the same time, HA acts as a protective shield against bacteria colonization [102,103]. Interesting data from an animal study suggested the inhibition of the downgrowth of connective tissue inside the peri-implant defect, facilitating bone regeneration and implant stability [104].
- Autologous platelet concentrates (APG) are promising biomaterials in periodontal and peri-implant regeneration. There are several protocols published (platelet-rich fibrin, PRF/A-PRF/L-PRF; platelet-rich plasma (PRP) platelet-rich growth factors, (PRGF) in the literature, and the main composition is based on platelet fibrin and growth factors, such as PDGF, vascular endothelial growth factors (VEGF), and transforming growth factors beta (TGF- b) [75,76,105]. They are defined as natural living cell scaffolds and according to several systematic reviews are valid biomaterials in periodontal and peri-implant regeneration [106,107,108] (Figure 8). The advantages are autologous origin and the fast and chip protocol. On the other hand, the handling and the production process differs among the types (PRF, A-PRF, PRP, PRGF). Another disadvantage is the fast resorption pattern that was estimated to be among 14 and 20 days [105]. Nevertheless, due to the fibrin scaffold and the presence of growth factors, they are promising biomaterial. Future studies are investigating PRF as a drug delivery system in periodontal defects [109].

## 3. Emerging Technologies

### 3.1. Stem Cell Therapies

Stem cells are cells of the human body capable of differentiating into any cell of an organism and are self-renewing. They are defined as unspecialized, and, in their evolution, there are various steps of specialization [110]. Research on cell-based approaches is concentrated on the use of mesenchymal stem cells (MSCs), multipotent stem cells with excellent biological proprieties obtainable from nearly all organs and tissues [111].

Periodontal ligament stem cells (PDL-SCs) are used in periodontal ligaments or cementum regeneration. They can be found in alveolar bone and root surfaces, though the PDL-SCs on the alveolar bone show better differentiation abilities. PDL-SCs can differentiate into mesenchymal cell lineages to generate adipocytes, collagen-forming cells, osteoblast-like cells, cementum tissue, and Sharpey’s fibers in vitro [110].

The discovery of periodontal ligament mesenchymal stem cells (PDL-MSCs) into the PDL proposes the important implication of them in the regeneration of the periodontium and its homeostasis. Although the use of PDL-MSCs on bone formation has provided contrasting results, the effect in increasing cementum and PDL formation seems to give good results. This capacity could be supported by the fact that PDL-MSCs express higher levels of various PDL-specific proteins than other MSCs [112]. From a clinical point of view, the use of stem cells is a promising adjuvant in the regenerative procedure. In any case, the limited availability and the requirement of a specialized laboratory render the use limited. In the last three years, a new concept was developed to facilitate the extraction through a mechanical process using simple handling directly in the dental office. Indeed, according to a previous review, this type of extraction seems to be promising in oral regeneration thanks to the combination with scaffolds as collagen membranes or grafts [111]. Studies on stem cells and innovative scaffolds show a potential improvement in term of periodontal regeneration [113,114]. Regarding the use in peri-implant regeneration, preclinical data showed promising results; nevertheless, further clinical studies are needed to validate their effect in peri-implant defects [115].

### 3.2. Three-Dimensional Printing

The introduction of 3D printing in the regenerative field enabled new bioresorbable polymers to be printed and customized for individual cases. The processes are several.

1. Inkjet model: consists of using inkjet printing with powder and liquid solutions to select and dispose of cells, create an extracellular matrix, and allows the use of a customized scaffold [116]. Park et al. (2012, 2014) published the use of a 3D fiber scaffold for guiding PDL cells and facilitating the mineralization of tissue [117,118]. Goh et al. (2015) analyzed the use of a 3D scaffold in socket preservation with normal bone healing and better-preserved volume [109].
2. Fusion model: allows building personalized scaffolds but without the inclusion of cells, growth factors, and proteins [116]. The polymer used is lactic-co-glycolic acid with good characteristics of resorption and mechanical strength.
3. 3D plotting allows the production of a soft scaffold composed of hydrogel with easy incorporation of cells. A limitation is the possible inhibition of cell-to-cell communication, influencing the signaling and proliferation process [119,120]. On the other hand, the use of living cells in the scaffold has great results for tissue formation.

## 4. Summary and Future Directions

**Figure 9:**
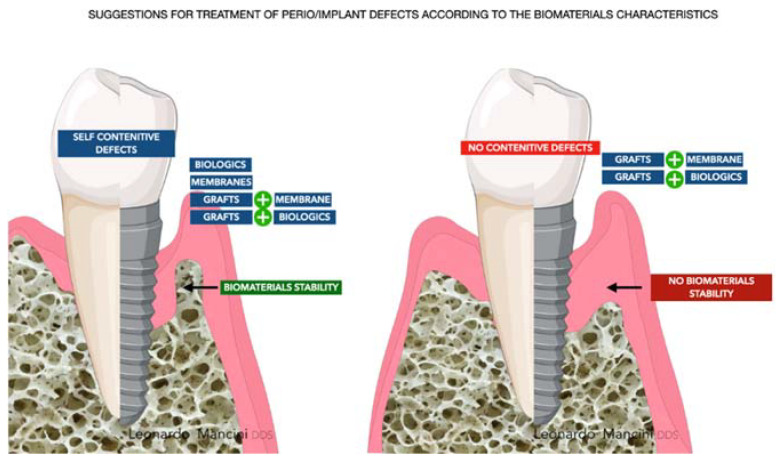
Biomaterials and their possible implications in periodontal/peri-implant regeneration.

The challenge for each periodontist is to restore all the components of the periodontal compartment (cementum, periodontal ligament, and bone). The regeneration around implants, from the tissue aspect, seems to be easier due to the regeneration of bone only. Nevertheless, it is still a crucial site due to the absence of the anatomical apparatus enriched with vessels, proteins, and growth factors located around teeth. The main elements that need to be considered to have a reliable regeneration are the managing of the occlusal load, mechanical stability of the biomaterial used (grafts better than biologics alone), the reduced FBR due to chemical and thermic treatments that allow the processing of particles, microbiological flora around the defects, dysbiosis control, and, lastly, wound stability. FBR should be discussed carefully due to possible failure related to exposure and infection of the grafts. Exposure is a crucial aspect in daily practice and in many, from an expected regeneration follow-up, has revealed the presence of fibrous encapsulation or graft rejection. The principles, mentioned before, are to be applied for every type of biomaterial and are at the basis of the regenerative process. Moreover, with the introduction of 3D biomaterials and the use of growth factors, signaling is another aspect that needs to be considered to recruit cells and guarantee a proper regenerative response around teeth or implants. However, the main targets are and will be the cost-effective and tailored approaches providing function and esthetics. According to this review for the question “Which is the best biomaterial?”, there is not a specific answer, but the synergetic potential of several biomaterials and the tailored approach lead to a reliable result and predictable regeneration (Figure 9).

## Figures and Tables

**Table 1:** Commercialized bone substitutes and heating temperature according to their production process and the manufacturing protocol.

Commercial Name	Sources	Heating Temperature
Bio-Oss^®^	Bovine	300 °C [38]
Re-bone^®^	Bovine	−80 °C to 121 °C [41]
Endobon^®^	Bovine	900 °C [42]
cerabone^®^	Bovine	1250 °C [43]
creos^TM^	Bovine	600 °C [44]
PepGen P-15^®^	Bovine	1100 °C [45]
SmartBone^®^	Bovine + Porcine	50 °C < [46]
Gen-Os^®^	Porcine	130 °C [47]
Zcore^®^	Porcine	500 °C to 620 °C [48]
THE Graft^TM^	Porcine	400 °C [49]
Equimatrix^®^	Equine	N/A
Bio-Gen^®^	Equine	130 °C [50]

**Table 2:** Collagen membranes classified according to the sources, main components, and cross-linking agent, as reported on the manufacturing instructions.

Commercial Name	Sources	Main Components	Cross-Linking Agent	Resorption Rate
Bio-Gide	Porcine	Type I and III collagen	None	24 weeks
Biostite	Calfskin	88% HA 9.5% type I collagen and 2.5% chondroitin sulfate	Diphenylphosphoryl azide	4–8 weeks
BioMend	Bovine	100% type I collagen	Formaldehyde	6–8 weeks
BioBar	Bovine	100% type I collagen	N/A	6–8 months
BioMend-Extend	Bovine	100% type I collagen	Formaldehyde	18 weeks
Periogen	Bovine	Type I and III collagen	Glutaraldehyde	4–8 weeks
Paroguide	Calfskin	96% type I collagen and 4% chondroitin sulfate	Diphenylphosphoryl azide	4–8 weeks
OsteoBiol	Equine	100% equine collagen	None	8 weeks
Tissue Guide	Bovine dermis + tendon	Atelocollagen + tendon collagen	Hexamethylene diisocyanate	4–8 weeks

## Data Availability

Data sharing not applicable. No new data were created or analyzed in this study. Data sharing is not applicable to this article.
